# Polyubiquitylation of AMF requires cooperation between the gp78 and TRIM25 ubiquitin ligases

**DOI:** 10.18632/oncotarget.1478

**Published:** 2013-10-28

**Authors:** Ying Wang, Seung-Wook Ha, Tianpeng Zhang, Dhong-Hyo Kho, Avraham Raz, Youming Xie

**Affiliations:** ^1^ Barbara Ann Karmanos Cancer Institute and Department of Oncology, Wayne State University School of Medicine, Detroit, MI, USA

**Keywords:** protein ubiquitylation, ubiquitin ligase, AMF, gp78, TRIM25

## Abstract

gp78 is a ubiquitin ligase that plays a vital role in endoplasmic reticulum (ER)-associated degradation (ERAD). Here we report that autocrine motility factor (AMF), also known as phosphoglucose isomerase (PGI), is a novel substrate of gp78. We show that polyubiquitylation of AMF requires cooperative interaction between gp78 and the ubiquitin ligase TRIM25 (tripartite motif-containing protein 25). While TRIM25 mediates the initial round of ubiquitylation, gp78 catalyzes polyubiquitylation of AMF. The E4-like activity of gp78 was illustrated by an *in vitro* polyubiquitylation assay using Ub-DHFR as a model substrate. We further demonstrate that TRIM25 ubiquitylates gp78 and that overexpression of TRIM25 accelerates the degradation of gp78. Our data suggest that TRIM25 not only cooperates with gp78 in polyubiquitylation of AMF but also gauges the steady-state level of gp78. This study uncovers a previously unknown functional link between gp78 and TRIM25 and provides mechanistic insight into gp78-mediated protein ubiquitylation.

## INTRODUCTION

Protein ubiquitylation plays a critical role in virtually all cellular processes [1-4]. Ubiquitylation is a multistep reaction involving three enzymes [5-7]. Ubiquitin (Ub) is first activated by the Ub-activating enzyme (E1), forming an E1~Ub thioester between the carboxyl group of the terminal glycine residue of Ub and the active site cysteine of E1. The activated Ub is thereafter transferred to a Ub-conjugating enzyme (E2), forming an E2~Ub thioester. With the participation of a Ub-ligase (E3), the Ub moiety of the E2~Ub thioester is conjugated, via an isopeptide bond, to the ε-amino group of a lysine residue on the target substrate. The donor Ub can also be added to a preceding Ub molecule (acceptor) attached on the substrate, resulting in a substrate-linked poly-Ub chain after successive rounds of conjugation. In several occasions, Ub is conjugated to the α-NH2 group of a substrate's N-terminal residue, a process called N-terminal ubiquitylation [8]. The substrate specificity is mainly determined by the Ub ligase, which binds both substrate and the E2 enzyme. Assembly of poly-Ub chains on some substrates can be facilitated by or needs cooperative interactions between two different Ub ligases [9-14]. In some cases, one of the two Ub ligases acts as an E4 enzyme, the so-called Ub chain elongation factor, which recognizes and elongates the nascent Ub chain (e.g., mono- or oligo-Ub chain) initiated by the other Ub ligase [9, 10]. In other cases, the functional relationship between pairs of Ub ligases is less clear.

gp78 is an endoplasmic reticulum (ER)-resident E3 Ub ligase that plays a vital role in ER-associated degradation (ERAD) [15-19]. The C-terminal domain of gp78 is exposed in the cytosol, which carries several functional subdomains, including a RING finger, a CUE (coupling of Ub conjugation to ERAD) domain, and a binding site for the Ube2g2 E2 enzyme. These subdomains are all essential to the E3 function of gp78 [16]. The known substrates of gp78 are diverse, including T cell antigen receptor subunits CD3*δ* and TCR*α* [15, 16], cholesterol metabolism regulatory proteins 3-hydroxy-3-methylglutaryl-CoA reductase, insulin-induced gene-1 and apoplipoprotein B [20-22], neurodegenerative disease proteins superoxide dismutase-1, ataxin-3 and mutant neuroserpin [23, 24], mutant cystic fibrosis transmembrane conductance regulator CTFR∆508 [12], cytochrome P450 3A4 [25], the Z variant of *α*1-antitrypsin [26], and the metastasis suppressor KAI1/CD82 [27]. Before the discovery of its E3 activity, gp78 was isolated as a cell surface receptor for the autocrine motility factor (AMF), and for this reason, gp78 is also known as AMFR [28, 29]. AMF is a moonlighting protein. When secreted outside tumor cells, it acts as a cytokine to promote cancer cell invasion and metastasis by stimulating cell motility upon binding to gp78 [30]. Inside the cell, AMF is equal to previously identified phosphoglucose isomerase (PGI) that catalyzes the interconversion of glucose 6-phosphate and fructose 6-phosphate [31]. Although the ligand-receptor relationship between AMF and gp78 has been established on cell surface, little is known about their interactions inside the cell.

In this study, we show that AMF binds to the cytoplasmic domain of gp78 and is a substrate of gp78. Remarkably, polyubiquitylation of AMF requires cooperation between gp78 and the Ub ligase TRIM25 (tripartite motif protein 25). We further demonstrate that TRIM25 regulates the metabolic stability of gp78. This study unveils a previously unknown link between gp78 and TRIM25 and presents an example of polyubiquitylation via cooperative interaction between two Ub ligases.

## RESULTS

### AMF binds the cytoplasmic domain of gp78

**Figure 1:**
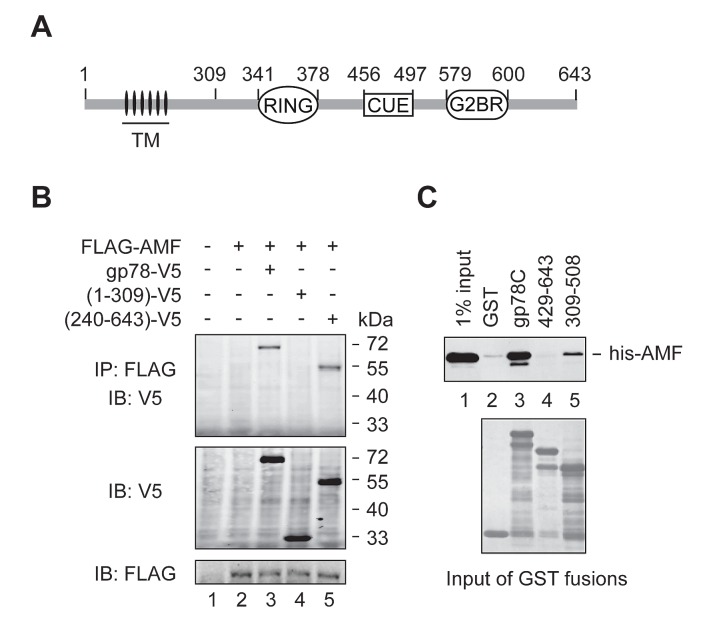
AMF binds the cytoplasmic domain of gp78 (A) Schematic representation of gp78. TM, transmembrane domains. (B) AMF is physically associated with gp78. N-terminally FLAG-tagged AMF was coexpressed with C-terminally V5-tagged gp78 or truncation mutants in HEK293T cells. Cell extracts were subjected to IP with a rabbit polyclonal anti-FLAG antibody, followed by immunoblotting with a mouse monoclonal anti-V5 antibody (top panel). The expression levels of gp78-V5 and FLAG-AMF were measured by immunoblotting with anti-V5 and anti-FLAG antibodies, respectively (middle and bottom panels). (C) AMF directly binds to the cytoplasmic domain of gp78. N-terminally his-tagged AMF expressed in and purified from *E. coli* was applied to pulldown assays with various GST fusions as indicated. Retained his-AMF was detected by immunoblotting with an anti-his antibody (top). Comparable input of GST fusions was confirmed by Coomassie blue staining (bottom).

To inspect the probable binding of AMF to the cytoplasmic domain (residues 309-643) of gp78 (Figure 1A), we first performed a co-immunoprecipitation (IP) assay. FLAG-tagged AMF was coexpressed with V5-tagged gp78 or truncated mutants in HEK293T cells. As shown in Figure 1B, full-length gp78 and the N-terminally truncated mutant gp78_240-643_ were co-immunoprecipitated with AMF (lanes 3 and 5). In contrast, the N-terminal fragment including the first 309 amino acids had no sufficient affinity for AMF (lane 4). We next used GST pulldown assay to examine whether AMF directly binds to the C-terminal domain of gp78. His-tagged AMF expressed in and purified from *E. coli* cells was incubated with GST fusions bearing the entire C-terminal domain of gp78 (gp78C) or truncated fragments. We found that AMF indeed directly interacted with the cytoplasmic domain of gp78 (Figure 1C, lane 3). Deletion of residues 309-428 of gp78 abolished the interaction with AMF (lane 4), and the fragment of residues 309-508 only retained a low affinity (lane 5), implying that an intact C-terminal domain is critical for the interaction with AMF.

### gp78 catalyzes polyubiquitylation of AMF

**Figure 2:**
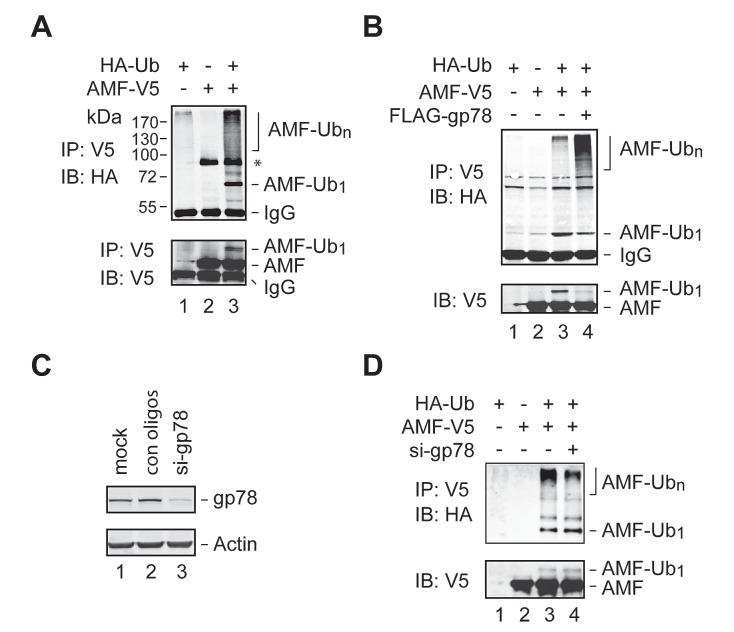
Polyubiquitylation of AMF by gp78 (A, B) Detection of ubiquitylated AMF. Cell extracts from HEK293T cells transfected with various expression vectors as indicated were subjected to IP with an anti-V5 antibody under nondenaturing (A) or denaturing (B) conditions, followed by immunoblotting analysis with an anti-HA antibody. The blot of A was reprobed with an anti-V5 antibody to position the monoubiquitylated AMF. A non-specific band (marked by an asterisk in A appeared in some but not other IP-immunoblotting assays. (C) Knockdown of gp78 expression by siRNA oligos. (D) Knockdown of endogenous gp78 impairs AMF polyubiquitylation. gp78-specific or control siRNA oligos were transfected into HEK293T cells coexpressing AMF-V5 and HA-Ub. Cell lysates were applied to IP with an anti-V5 antibody, followed by immunoblotting with an anti-HA antibody. Input of cell extracts was examined by immunoblotting with an anti-V5 antibody.

To examine if AMF is ubiquitylated in the cell, we coexpressed V5-tagged AMF (AMF-V5) and HA-tagged Ub (HA-Ub) in HEK293T cells. Cell extracts were subjected to IP with an anti-V5 antibody, followed by immunoblotting analysis with an anti-HA antibody. Ubiquitylated AMF including poly- and mono-ubiquitylated species were readily detected (Figure 2A, top panel). The monoubiquitylated species of AMF was further confirmed by reprobing the blot with an anti-V5 antibody (bottom panel). To ensure that the proteins detected were AMF rather than other ubiquitylated proteins coimmunoprecipitated with AMF, we repeated the IP experiment using cell extracts prepared under denaturing conditions. As shown in Figure 2B, AMF was indeed ubiquitylated. This assay also revealed that overexpression of gp78 increased the polyubiquitylation of AMF (compare lanes 3 and 4). We next examined if ubiquitylation of AMF is dependent on gp78. Specific siRNA oligos were used to reduce the expression of endogenous gp78 (Figure 2C). We found that polyubiquitylation of AMF was substantially reduced by knockdown of gp78 (Figure 2D, compare lanes 3 and 4). Taken together, these results demonstrate that gp78 catalyzes polyubiquitylation of AMF.

### TRIM25 mediates initial ubiquitylation of AMF

**Figure 3:**
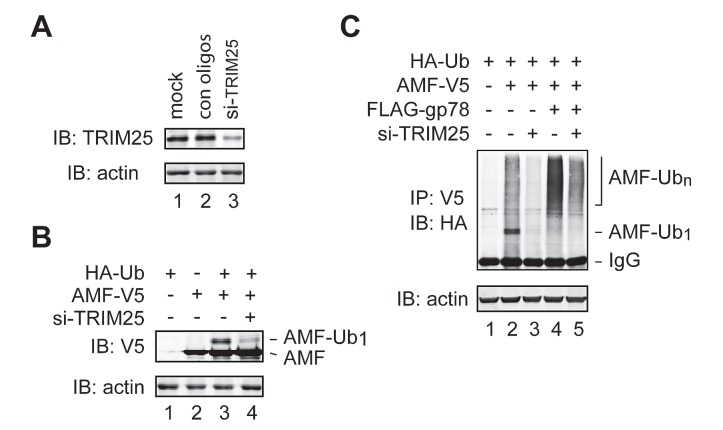
TRIM25 is required for initial ubiquitylation of AMF (A) Knockdown of TRIM25 expression by siRNA oligos. (B) TRIM25 is required for initiation of AMF ubiquitylation. TRIM25-specific or control siRNA oligos were transfected into HEK293T cells coexpressing AMF-V5 and HA-Ub. Monoubiquitylated AMF was detected by immunoblotting with an anti-V5 antibody. (C) Knockdown of TRIM25 inhibits polyubiquitylation of AMF. TRIM25-specific or control siRNA oligos were transfected into HEK293T cells coexpressing AMF-V5 and HA-Ub without (lanes 2 and 3) or with (lanes 4 and 5) overexpression of gp78. Ubiquitylation of AMF was measured by IP-immunoblotting analysis.

Of note, although knockdown of gp78 impaired AMF polyubiquitylation, it did not inhibit but modestly increased monoubiquitylation of AMF (Figure 2D). In addition, the increase of polyubiquitylation of AMF by overexpression of gp78 was concomitant with a decrease of monoubiquitylated AMF (Figure 2B). These observations indicate that gp78 is not required for the first round of AMF ubiquitylation. It may target and catalyze further ubiquitylation of monoubiquitylated AMF. Then, what is the E3 enzyme that initiates AMF ubiquitylation? In search for the relevant E3 enzyme, we used IP-coupled liquid chromatography-tandem mass spectrometry (LC-MS/MS) to identify novel AMF-associated proteins. One of the proteins thus isolated turned out to be TRIM25 (also called estrogen-responsive finger protein or EFP), a member of the tripartite motif protein family [33, 34]. It has been shown that TRIM25 is a Ub ligase and plays a critical role in cell cycle regulation, immune signaling, and antiviral protection [35-37]. We went on to examine whether TRIM25 was required for initial ubiquitylation of AMF. Specific siRNA oligos were used to reduce the expression of endogenous TRIM25 (Figure 3A). The amount of monoubiquitylated AMF markedly decreased upon knockdown of TRIM25 (Figure 3B, compare lanes 3 and 4, Figure 3C, compare lanes 2 and 3). As a consequence, polyubiquitylation of AMF was severely impaired (Figure 3C, compare lanes 2 and 3). The effect of gp78 overexpression on AMF polyubiquitylation was also diminished by knockdown of TRIM25 (Figure 3C, compare lanes 4 and 5). These results indicate that TRIM25 acts upstream of gp78 to initiate AMF ubiquitylation and suggest that gp78 may function as an E4-like enzyme that elongates the Ub chain on AMF.

### gp78 is an E4-like enzyme

**Figure 4:**
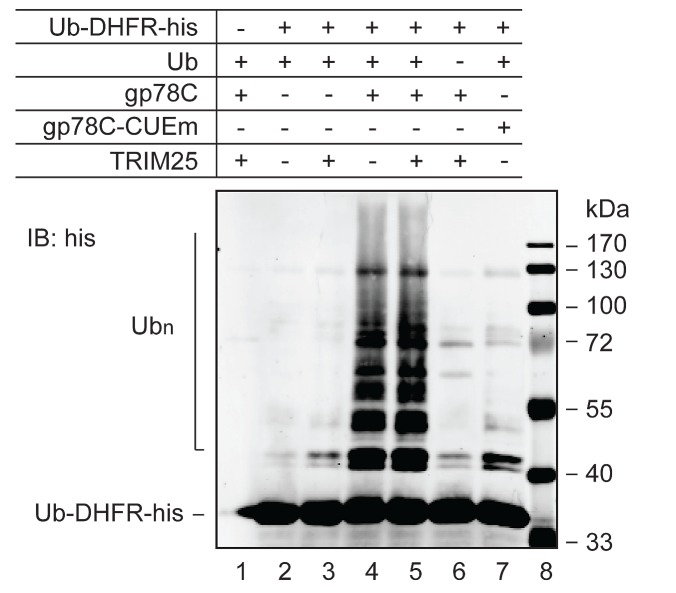
E4-like activity of gp78 In vitro ubiquitylation reactions were set up with purified proteins as indicated to measure polyubiquitylation of Ub-DHFR-his. UBE1 (E1) and UBC5 (E2) were included in all reactions. The reactions lasted for 90 min and were terminated by addition of sample buffer. Proteins were resolved on SDS-PAGE and analyzed by immunoblotting with an anti-his antibody.

Our attempts to recapitulate the role of TRIM25 and gp78 *in vitro* using AMF purified from *E. coli* in a reconstitution system have been unsuccessful. It is possible that AMF needs to be modified prior to recognition and ubiquitylation by TRIM25 and the posttranslational modification is lacking in *E. coli*. A previous work showed that gp78 was able to catalyze polyubiquitylation of Ub-GST, a model substrate that mimics monoubiquitylated GST [12]. This observation suggested that gp78 might have an E4-like activity. However, it is not clear from this previous study whether the E4-like activity was contributed by gp78 or came from gp78-associated proteins because gp78 was isolated from gp78-overexpressing HEK293 cells through one-step immunoprecipitation. To more directly determine if gp78 possesses E4-like activity, we purified GST-gp78C from *E. coli*, which lacks the ubiquitin system. It has been shown that GST-gp78C retains the Ub ligase activity [16]. We tested if GST-gp78C could polyubiquitylate the Ub-DHFR model substrate [38]. A CUE domain mutant (GST-gp78C-CUEm) defective in Ub binding served as a negative control. A his tag was added to the C-terminus of Ub-DHFR for purification and immunoblotting analysis. As shown in Figure 4, GST-gp78C but not GST-gp78C-CUEm polyubiquitylated Ub-DHFR-his (compare lanes 4 and 7). This result demonstrates that gp78 is an E4-like enzyme. Of note, TRIM25 neither catalyzed ubiquitylation of Ub-DHFR-his (lane 3) nor enhanced the activity of gp78 (compares lanes 4 and 5). These observations are in line with the role of TRIM25 in the initiation of AMF ubiquitylation.

### gp78 is a substrate of TRIM25

**Figure 5:**
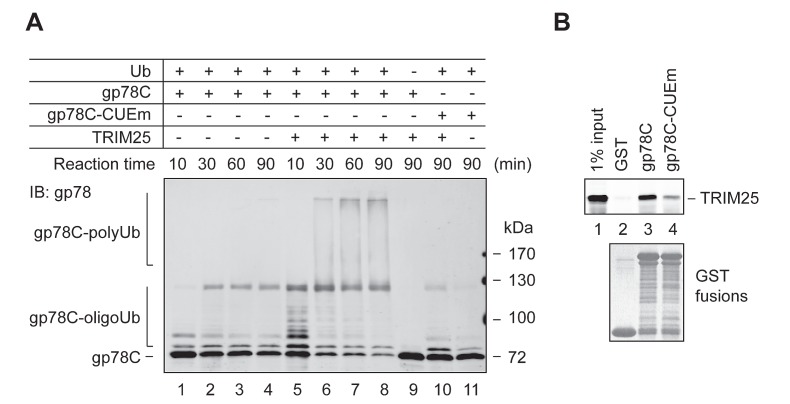
gp78 is a substrate of TRIM25 (A) In vitro ubiquitylation of GST-gp78C by TRIM25. An in vitro time course ubiquitylation assay was set up to compare ubiquitylation of GST-gp78C in the absence (lanes 1-4) or presence (lanes 5-8) of TRIM25. GST-gp78C-CUEm was used as a control (lanes 10, 11). A reaction lacking Ub served as a negative control (lane 9). (B) TRIM25 binds to the cytoplasmic domain of gp78. Purified TRIM25 protein was incubated with GST-gp78C, GST-gp78C-CUEm, or GST. Retained TRIM25 was detected by immunoblotting with an anti-TRIM25 antibody.

In the course of testing in vitro ubiquitylation of AMF by TRIM25 and gp78, we found that TRIM25 was able to ubiquitylate gp78. This was illustrated by a time course ubiquitylation reaction (Figure 5A). Specifically, we compared the ubiquitylation of GST-gp78C in the presence or absence of TRIM25 in a reconstitution system. As expected, GST-gp78C displayed intrinsic autoubiquitylation activity, forming oligoubiquitylated species (lanes 1-4). However, no polyubiquitylated GST-gp78C was detected after 90 min of the reaction. In contrast, substantial amounts of GST-gp78C were readily polyubiquitylated after 30 min of the reaction in the presence of TRIM25 (lanes 5-8). Oligoubiquitylation of GST-gp78C was also more efficient in the presence than in the absence of TRIM25 (compare lanes 1 and 5). Note that TRIM25 did not polyubiquitylate GST-gp78C-CUEm under the same conditions (lane 10). This is likely because TRIM25 has a much lower affinity for GST-gp78C-CUEm than GST-gp78C, as shown in the GST pulldown assay (Figure 5B). Taken together, these results indicate that gp78 is a substrate of TRIM25.

**Figure 6:**
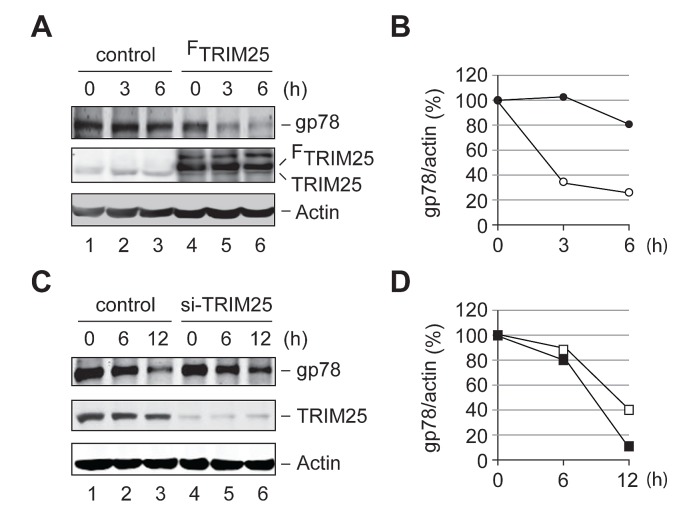
TRIM25 accelerates the metabolic degradation of gp78 (A) Degradation of gp78 is enhanced by overexpression of TRIM25. CHX chase analysis was carried out to compare the turnover of endogenous gp78 with (lanes 4-6) or without (lanes 1-3) overexpression of FLAG-tagged TRIM25 (^F^TRIM25). (B) Quantification of data in A. The Odyssey infrared imaging system was used to quantify the intensity of gp78 and actin bands. The relative ratio of gp78 to actin was plotted as a function of the chase time. Open circle, ^F^TRIM25; filled circle, control vector. (C) Knockdown of TRIM25 slows down degradation of gp78. The effect of knockdown of TRIM25 on the metabolic stability of gp78 was measured by the CHX chase analysis. (D) Quantification of data in C. The gp78 decay curves were plotted as B. Open square, si-TRIM25 oligos; filled square, control oligos.

We next examined whether TRIM25 affects the metabolic stability of gp78 in the cell. To this end, we carried out a cycloheximide (CHX) chase assay to compare the degradation of endogenous gp78 in HEK293T cells with or without overexpression of TRIM25. We found that overexpression of TRIM25 accelerated the turnover of gp78 (Figures 6A and B). On the other hand, knockdown of TRIM25 slowed down the degradation of gp78 (Figures 6C and D). Thus, TRIM25 influences the metabolic degradation of gp78 in the cell.

## DISCUSSION

In this study, we demonstrate that ubiquitylation of AMF requires two Ub ligases, gp78 and TRIM25. The first round of ubiquitylation is mediated by TRIM25, whereas gp78 acts as an E4-like factor to catalyze polyubiquitylation of AMF. Of note, although gp78 directly interacts with AMF, it is unable to initiate the ubiquitylation of AMF. Perhaps, the ubiquitylation site of AMF may be too far from the active center of gp78, i.e. the E2~Ub thioester associated with gp78. Conjugation of the first Ub molecule by TRIM25 may shorten the distance, allowing transfer of donor Ub from the gp78 active center to the preceding Ub molecule (acceptor Ub) on AMF. Similar to our observations, a previous study has shown that gp78 cooperates with the RMA1 E3 enzyme to ubiquitylate CFTR∆508 [12]. It will be of great interest to revisit the ubiquitylation of other known gp78 substrates to determine if gp78-catalyzed polyubiquitylation generally requires an additional E3 enzyme and if gp78 pairs with different E3 enzymes for different substrates.

Cooperative interactions between pairs of Ub ligases in protein ubiquitylation have recently been reported for some other substrates. For example, Ufd2 cooperates with Ufd4 to ubiquitylate UFD (Ub-fusion degradation) substrates [9]. Ubiquitylation of p53 requires the collaboration of MDM2 with CBP or p300 [13, 14]. Ufd4 can enhance Ubr1-initiated ubiquitylation of N-end rule substrates [11]. In some cases, one of the two Ub ligases acts as an E4 enzyme that, by definition, recognizes and elongates the nascent Ub chain (e.g., mono- or oligo-Ub chain) initiated by the other Ub ligase. The yeast Ufd2 is the first identified and by far the best characterized E4 [9, 10]. In other cases, the functional relationship between two Ub ligases is less clear. The activity of one Ub ligase depends on initial ubiquitylation by another E3, but it is unclear whether this Ub ligase polymerizes the very same nascent Ub chain. These Ub ligases are considered a special class of E3 enzymes, which possess E4-like activity but remain to be determined whether they are a typical E4 enzyme. It remains to be determined whether gp78 is a classic E4 enzyme.

It has been shown that TRIM25 plays a critical role in inducing interferon production in response to viral infection via ubiquitylation of RIG-1 [35]. TRIM25 also functions as an ISG15 E3 ligase that targets 14-3-3σ for ISGylation [36, 37]. The current work reveals that TRIM25 not only initiates the ubiquitylation of AMF, but also ubiquitylates gp78. The dual activity of TRIM25 is biologically significant as it provides a balance act to regulate polyubiquitylation of AMF. On the one hand, TRIM25 generates the preferred substrate (e.g., monoubiquitylated AMF) for gp78; on the other hand, it enhances the metabolic turnover of gp78. To our best knowledge, this study for the first time reveals that a Ub ligase can regulate ubiquitylation mediated by another Ub ligase by controlling the availability of the substrate and the Ub ligase. It will be of interest to investigate the potential role of TRIM25 in gp78-mediated ERAD.

## MATERIALS AND METHODS

### Cell culture and reagents

HEK293T cells were grown in Dulbecco's modified Eagle's medium containing 10% fetal bovine serum and maintained at 37°C in a humidified atmosphere of 5% CO_2_ and 95% air. Transfection was performed with FuGENE HD transfection reagent (Roche) according to the manufacturer's instructions. Full-length human AMF cDNA generated by PCR was inserted into pCDNA6-V5, pCDNA3-FLAG-B or pCDNA6-V5/His-B (Invitrogen) at the EcoRI and XbaI sites. Full-length human gp78 cDNA was inserted into pCDNA6-V5 or pCDNA3-FLAG-B at the BamHI and XbaI sites. Deletion mutants of gp78 were constructed by subcloning PCR-amplified fragments into pCDNA6-V5. All constructs were verified by DNA sequencing. The plasmid expressing N-terminally HA-tagged Ub (HA-Ub) was a gift from D Bohmann [32]. The *E. coli* expression vectors of GST fusions with gp78 C-terminal fragments or CUE domain mutant were kindly provided by A Weissman [16]. The resources of antibodies used in this study were as follows: mouse monoclonal anti-V5 (Invitrogen), anti-HA and anti-*β*-actin (Sigma-Aldrich), anti-FLAG (Origene), anti-AMF (Thermo Scientific Dharmacon), anti-TRIM25 (BD Biosciences) and anti-gp78 (Abnova), rabbit polyclonal anti-AMF and anti-his (Santa Cruz Biotechnology), anti-gp78 (Bethyl Labs) and anti-FLAG (Sigma-Aldrich). gp78 and TRIM25 specific siRNA oligos were purchased from Invitrogen and Thermo Scientific Dharmacon, respectively.

### Immunoblotting and IP assays

Cells were harvested and lysed with RIPA buffer (50 mM Tris-HCI, pH 7.5, 150 mM sodium chloride, 1.0% NP-40, 0.5% sodium deoxycholate, 0.1% sodium dodecyl sulfate) containing protease inhibitor cocktail for 20 min at 4 °C. The lysates were centrifuged at 14,000 rpm at 4 °C for 15 min to remove debris. Protein concentrations were determined using the Bio-Rad Protein Assay kit. For immunoblotting analysis, 50 μg of cell lysates were separated by SDS-PAGE and transferred to polyvinylidene fluoride membranes (Millipore). Membranes were blocked in 0.2% PBS containing 0.1% casein for 1 h, and then incubated with appropriate primary antibodies for 2 h. After 3 washes, membranes were incubated with secondary antibodies conjugated with IRDye 800 (Rockland Immunochemicals) or Alexa Fluor 680 (Invitrogen) for 40 min. The immunoblots were visualized using an Odyssey Infrared Imaging system (LI-COR Biosciences). For IP experiments, 500 μg of cell lysates were incubated with antibody for 1 h at 4°C, followed by 1 h incubation with Protein A/G agarose beads. The beads were then washed extensively with RIPA buffer and incubated with sample buffer at 95°C for 3 min. Eluted samples were subjected to SDS-PAGE, followed by immunoblotting analysis. To perform IP assays under denaturing conditions, cell extracts were treated with 2% SDS at 95°C for 2 min, clarified by centrifugation and diluted with RIPA buffer (without SDS) to the final concentration of SDS to 0.1%. The lysates were then subjected to IP with anti-V5 antibody, followed by immunoblotting analysis with anti-HA antibody. For the detection of AMF ubiquitylation, MG132 was added to the culture medium at a final concentration of 2 mM 12 h before harvesting cells. For co-IP experiments, cells were lysed in buffer A (50 mM Tris-HCl, pH 7.5, 150 mM NaCl, 0.5% NP40) containing protease inhibitor mixture. Clarified cell extracts were incubated with the primary antibody for 1 h at 4°C, followed by 1 h incubation with Protein A/G agarose beads. The beads were then washed extensively with buffer B (50 mM Tris-HCl, pH 7.5, 150 mM NaCl, 0.1% Triton X-100). To prepare samples for LC-MS/MS, extracts from HEK293T cells overexpressing C-terminally V5-tagged AMF were loaded on anti-V5-coupled magnetic beads. The immunocomplex containing AMF-V5 was resolved by SDS-PAGE. All protein bands except for AMF-V5 were subjected to LC-MS/MS.

### CHX chase assay

After 24 h of overexpression or knockdown of TRIM25, CHX (100 μg/ml) was added to the culture medium to stop protein synthesis. Aliquots of cells were withdrawn at different time points. Endogenous gp78 protein was immunoprecipitated by a rabbit polyclonal anti-gp78 antibody, followed by immunoblotting with a mouse monoclonal anti-gp78 antibody. Lysate input was examined by immunoblotting with an anti-actin antibody. The relative ratio of gp78 vs. actin was plotted as a function of the chase time to construct the gp78 decay curves.

### Protein purification

Expression of GST fusion proteins, his-AMF, and Ub-DHFR-his in *E. coli* (BL21) was induced by 0.3 mM isopropyl β-D-1-thiogalactopyranoside (IPTG) for 3 h at 25 °C. Cells were harvested and resuspended in buffer C (50 mM HEPES, pH 7.5, 150 mM NaCl, 1 mM EDTA, and 0.2 % NP-40) supplemented with 0.1 mg/ml of lysozyme and protease inhibitor cocktail. Note that EDTA was excluded from buffer C for purification of his-AMF and Ub-DHFR-his by nickel beads. Cells were lysed by ultrasonication and centrifuged at 13,200 rpm for 10 min at 4°C. The supernatants were further clarified by ultracentrifugation at 100,000 rpm for 1 h at 4°C. To purify GST fusion proteins, cell extracts were loaded onto 0.5 ml glutathione-agarose column equilibrated with buffer C, followed by washing with 20 ml of buffer C. GST fusion proteins were then eluted by 10 mM of reduced glutathione in 50 mM Tris-HCl, pH7.5. For purification of his-AMF and Ub-DHFR-his, cell extracts were applied to 0.5 ml Ni-NTA agarose column equilibrated with buffer C (minus EDTA) containing 20 mM imidazole, followed by washing with 20 ml of equilibration buffer. his-AMF and Ub-DHFR-his proteins were then eluted by step-gradient of imidazole from 60 to 200 mM in 50 mM Tris-HCl, pH7.5. After desalting through PD-10 column (GE healthcare), fractions were concentrated by ultrafiltration (10 kDa MWCO, Millipore).

### GST pulldown assay

The amounts of GST fusion proteins used in the pulldown assays were determined experimentally. An aliquot of lysate was applied to 10 μl of glutathione-agarose in 500 μl of buffer C and incubated for 1 hr at RT. The unbound proteins were removed by 3 washes with buffer C and then equilibrated with buffer D (50 mM Tris/HCl, pH 7.5, 100 mM NaCl, 1 mM DTT, and 0.1 % Tween20). The equilibrated beads were incubated with purified his-AMF or TRIM25 (OriGene Technologies) with gentle rolling for 3 hr at 4 °C. After removing unbound proteins by 3 washes with buffer D, the bound proteins were eluted by SDS-PAGE sample buffer, separated on SDS-PAGE, and examined by immunoblotting with an anti-AMF or anti-TRIM25 antibody.

### In vitro ubiquitylation assay

A typical in vitro ubiquitylation assay was conducted in a 10 μl volume of reaction containing 100 nM UBE1, 2 μM UBC5, 50 μM ubiquitin (Boston Biochem), and 0.25 μg of purified Ub-DHFR-his or his-AMF in buffer E (25 mM HEPES, pH 7.8, 25 mM KCl, 0.25 mM DTT, 5 mM MgCl2, 4 mM ATP). Depending on different experiments, 0.25 μg of GST-gp78C, GST-gp78C-CUEm and 100 nM TRIM25 were added individually or in combination into the ubiquitylation reactions, which were continued for up to 90 min at 37 °C and then terminated by SDS-PAGE sample buffer. Proteins were resolved on SDS-PAGE, followed by immunoblotting analysis with anti-AMF, anti-his, or anti-gp78 antibody.
